# ‘Which Piece Fits in Precisely Where?’: Disorientation as Queer Strategy in Ann Quin’s *Three*

**DOI:** 10.1080/09574042.2025.2469999

**Published:** 2025-05-20

**Authors:** Alice Hill-Woods

**Keywords:** Queer theory, avant-garde, experimental, temporalities, British fiction

## Abstract

The British experimental writer, Ann Quin (1936–73), has gained notoriety in recent years for her unconventional prose that decidedly swims against the current of canonical writing from the time. While she lived a life that strayed beyond heteronormative ideals, scholars rarely categorize her as a queer author. Her fragmentary novel *Three* (1966) follows a tripartite relationship between a couple and their young female lodger, and its plot and form leave much to be queried. Taking an approach informed by queer theory, this article positions *Three* as a distinctly queer novel, using disorientation as a lens for reading Quin’s experimentation with language. Mediated by its evocatively arranged garden objects, temporal flux, disorientating losses and gaps, and utopian dreams and reveries encoded in complex syntax, *Three* operates as a text with plentiful potential. *Three* calls forth both the uncomfortable and painful dimensions of nonnormative desires, as well as making clear what ambitious experiments in fiction can do: that is, propose a different, more queer-tender world.

In an interview with John Hall for the *Guardian* in April 1972, Ann Quin described her bisexual experience as something that ‘was so far beyond the fantasy that [she] found it very, well, you could say enlarging’.^1^ Yet critics rarely consider her to be a queer author, and her novels are infrequently read through a queer lens. Quin’s slippery language has, in recent decades, had a hypnotic effect on her committed readership, to the extent that Nonia Williams recommends exercising caution when ‘recovering’ the writer through archival matter and other references to her life, which regularly inspire a sense of intimacy.^2^ Is it the oddness of her prose that draws readers to this sense of secret familiarity, like an acquired taste, or the knowledge that her writing impulse prevailed despite a life full of setbacks? The seductive pull of Ann Quin reveals glimpses of perversity, the refusal of rules, and what Hilary White calls the ‘irreal’ quality of her writing.^3^ Even the reasons behind her death by drowning in 1973 escape any watertight conclusion, and so those who outlive her meander through, and try to make sense of, the residues of her weirdness. Examining *Three* (1966), the second of Quin’s four novels before her death, I consider disorientation as a critical tool in its construction. *Three* invites contemplation; Adam Guy, for example, attends to *Three*’s ‘auralities’ and contributes to the project of careful recuperation and ‘retrieval’ to foreground the novel’s resonances with the medium of tape recording.^4^ Alice Butler examines the triangular shape of desire in Quin’s *Three* and its ‘radical geometries’, offering a reading that pays attention to the novel’s affections while reflexively drawing upon her own tenderness towards another writer involved in the Quin fixation.^5^

It is my own fixation, as it were, that leads me to consider disorientation as a way of reading Quin’s texts, whereby her dissociative experiments in language are not indicative of mental illness or psychological crisis, but rather a queering of the avant-garde imaginary. I approach disorientation as an umbrella term, which I then employ as a means of tracing the queer dimension in Quin’s textual practice. This sheds light on *Three*’s objects of desire, its troubled fabric of reproductive time, its structural puzzles and its semantic potential. Read in light of queer theory, Quin’s disorientations lead us towards imagining what a queer telos might look like in language, in structure, and in what is felt and experienced. In its broader context, this reading of *Three* contributes to the project of queering the British 1960s. Patricia Juliana Smith asserts that, alongside significant historical landmarks such as the Stonewall Riots in 1969, queer culture ‘flourished in [the United Kingdom and the United States of America] with an elaborate and highly developed sensibility, a subcultural vernacular and semiotic system’.^6^ In keeping with Smith’s claim, my analysis of *Three* parses the strategies employed by a queer, subcultural author for an audience reading at the turn of socio-political transformation.^7^ Queer writing of this time took risks; Eveline Kilian, for example, views the structural mosaics in Maureen Duffy’s *The Microcosm* (1966) as ‘probing and destabilizing established gender norms and expectations’ in alignment with Duffy’s queer activism, while Len Gutkin reads camp in Brigid Brophy’s *In Transit* (1969) as ‘gender performance and sex mutability’.^8^ Reading queerness in Quin’s writing thus opens it up to different kinds of textures and disruptions.

*Three* is an assemblage of distortions and relations; its temporal fabric is elastic, and its prose is surreal in some places and mundane in others. The narrative begins proleptically, after the death of S, a younger lodger living in a household that belongs to Ruth and Leonard, a childless, married couple. The cause of S’s death is uncertain, although suicide is suggested at the outset. Ruth and Leonard piece together fragments of the life she left behind in the form of recollections, tape recordings and diary entries, all of which gesture, retrospectively, to the trio’s ambiguous relationship. In this way, *Three* achieves much of its narrative thrust through the characters’ perpetual mediation between the past and the present. The narrative is not a complete abstraction, however; there are indexes of standardized time, such as:

October 15th Clear day. Sun at last. S hasn’t returned.

October 16th Rain again. Still no sign of S. Informed police.

October 18th Boat found capsized. Coat identified. Also note in pocket – looks like suicide.^9^

Yet this façade of linear time only seems to exacerbate the text’s lack of temporal anchoring. The text omits some punctuation, such as inverted commas, despite its reliance upon dialogue between Ruth and Leonard. As a consequence, the narrative is shaped by a pronounced slippage between identities, whereby each character’s voice becomes difficult to identify. It is the first sign of disorientation as a narrative strategy.

Examining the ways in which a narrative implements (dis)orientating strategies pulls queer studies and temporal theory into close proximity. Ahmed writes that ‘orientations shape not only how we inhabit space, but how we apprehend this world of shared inhabitance, as well as “who” or “what” we direct our energy and attention toward’.^10^ Ahmed contends that the first steps towards ‘queer phenomenology’ might consider objects that are ‘less proximate’ or ‘deviant’, to the extent that such objects mediate the sense of being (dis)orientated.^11^ Quin’s settings are suffused with objects; readers glimpse Ruth’s anxious temperament in the way ‘she straighten[s] cushions, place[s] objects in different positions, replace[s] chairs, slide[s] the doors apart, [stands] between, and face[s] the room’, suggesting that she straightens out the domestic space to affirm the presence of normativity in a life that has become subject to significant queering; Butler reads this as a gendered ‘emotional frenzy’ which has become habituated over time.^12^ It becomes evident that objects encountered in *Three* can be perceived as significant to both queerness and temporality, striking examples of which appear in Ruth and Leonard’s garden, the mise-en-scène of the trio’s queer debauchery.

## Queer Garden Objects

Quin’s phenomenological narratives are significant because they provoke a particular voyeurism in the reader, where every potentially unremarkable object is presented remarkably not only in its aesthetic construction but also by means of its affective connotations. This enables Quin to present minutiae in intimate, aslant probes, enhancing the reader’s proximity to details within her compositions. These details are, as Philip Stevick suggests, ‘almost inevitably sexually charged’.^13^ Yet they are also strongly indicative of queer temporalities. Leonard and Ruth’s garden is attached to ‘The Grey House’, which is ‘off-white, appertaining to a Georgian era, but built in the early twenties’, an early example of the novel’s figuring of anachronism.^14^ Beyond the house, garden matter mediates the narrative’s complex relation to time. The tomatoes, for example, appear to resist any organic temporal pull; Ruth ‘wonders why the tomatoes she looks at every day never go beyond being green or yellow’.^15^ Leonard’s orchids, on the other hand, represent a fertile time and space, located within the summerhouse which suspends the heat of the season with its perfect horticultural conditions. As if to further distort the garden’s abnormal tonalities, Leonard’s devotion to his orchids is deeply sexual:

A bee orchid leaned over from the moistness around, touched his mouth. The petals fluttered. He put the pot on another shelf. Touched others. Slits of brilliant colour amongst a mass of green dripping foliage. Roots emerged suddenly as he parted leaves. Thrust through. He poked about with his little finger. He murmured with pleasure, sometimes sighed. Leaves sprang back into place. Tips of red and purple dipped, shrivelled into the dampness. […] Paused longer at some, peered into centres, ran a finger along stems, pink against pink laid there*.*^16^

Quin’s figurative language of movement, moisture and hue paints the orchids in fleshy, animated overtones, reminiscent of aroused genitalia; her hypersexual language of flowers deviates from the prudish conventions of domestic gardening. In contextual terms, the notion of a domestic garden is rife with cultural meaning. Jack Goody, in *The Culture of Flowers* (1993), compares the ‘garden of orientation (birth)’ with the ‘garden of procreation (marriage)’ and the ‘garden that one establishes as a householder’, implicating these sites with linear passages of heteronormative time.^17^ He posits that flowers are culturally entwined with the ‘human cycle of birth, marriage and death’.^18^ Suggestive of extramarital pleasure, then, Leonard’s seductive orchids signify a queering of heteronormative time.^19^ The garden offers an ecological mapping of disorientation through its erotic departure from conventions.

This aesthetic is further exacerbated by Quin’s reference to statues, which furnish the space as potentially erotic objects and, like the orchids, are given a degree of animacy:

L brought some of the statues down, arranged into rows, facing the platform. We mimed, sang, danced between, flinging some flowers, old clothes over the bronze and metal pieces of sculpture, a few without heads. […] Sometimes just the two of us, exchange masks, hide behind the statues, pretend making love to them, and L’s laugh loud, louder until darkness drives us out, back to the house.^20^

This passage conveys a performative, ritualistic intimacy between the characters, whereby the statues, which ‘seem human’, are positioned mimetically in order to mirror the queer relationship forming between the trio.^21^ Statues such as ‘Hallucination Aphrodite’ are broken, renamed and used in theatrical jest, parodying a time generative of classical antiquity.^22^ They also function as objects that blur gender boundaries through their deliberate misshapenness. Ruth laments that the Aphrodite figure is ‘so grotesque’ in that it ‘didn’t even have a head’, speculating: ‘was it a man or a woman that thing sticking out of what looked like breasts’.^23^ Julia Jordan interprets this ambivalence as affirming a ‘late-modernist ontological ambiguity’, stressing that *Three*’s focus on ‘objects that don’t fit anywhere; fragments of memory that don’t make sense; particles of matter that have become displaced’ are indicators for ‘the accidental nature of life’.^24^ Although Jordan suggests that these displacements are exemplary of broader, avant-garde experiments, her observations echo Josh Powell’s suggestion that Quin’s characters display behaviour characteristic of a ‘schizoid condition’, demonstrated by their frequent psychic dissociations and material dislocations.^25^ Moreover, Jordan cites R. D. Laing’s theory of ontological insecurity which, in turn, frames Quin’s characters as psychologically unstable; ontological insecurity occurs when ‘the world of [an individual’s] experience comes to be one [they] can no longer share with other people’.^26^ While Quin indeed depicts the statues as physically disordered and ontologically disorientating, this exemplifies Quin’s lively figuring of queerness rather than illness.

*Three*’s disorientating object relations are not symptomatic of a split self, but rather an emergent queer self. In phenomenological terms, the garden objects resemble the kind of ‘found objects’ generative of ‘the place where human desire manifests itself’, to borrow a phrase from Maurice Merleau-Ponty.^27^ Returning to Ahmed’s argument, it becomes increasingly useful to query how scenes of disorientation can shape and illuminate modes of existence that are otherwise obscured by heterocentric readings.^28^ Ahmed explores whether such disorientations ‘can offer us the hope of new directions, and whether new directions are enough for hope’.^29^ In a likewise optimistic inquiry, rather than casting Quin’s objects as symbols for psychic disconnectedness, a queer reading reveals the opposite: Leonard, Ruth and S strive for meaningful connections with each other, acting out their desires through ludic object encounters and fantasy-making. S’s latent desires materialize belatedly in her confessions. She writes: ‘feelings—feelings? What would it mean to them what has been what will be?’, divulging the protracted duration of her queer feelings in the present by denoting the past (‘what has been’) and the future (‘what will be’).^30^ Her capacity to act out these feelings is somewhat limited to the garden, which thus becomes a theatre for queer potential. As José Esteban Muñoz argues, on ‘various theatrical stages […] and arenas both subterranean and aboveground, queers live, labor and enact queer worlds in the present’.^31^ Muñoz’s notion of ‘the future in the present’ considers how embodied performances ‘contain […] an anticipatory illumination of a queer world, a sign of an actually existing queer reality’.^32^ In this light, the garden performances in *Three* exist within a time–space schema wherein experimental queer fantasies can be rehearsed, buttressed by the interplay of deviant object-props. The performances become a means of pulling past, present and future desires into an integrated display, culminating in a thorough ‘testing out’ of queer relations.

## Disorientation and Discomfort

*Three*’s queer encounters rely on variants of space to flesh out their motives, but this is not always optimistic. As a novel that deals initially with death, loss and violence, *Three* is bound to negative affective representations. Disorientation can be profoundly uncomfortable; a ‘feeling of shattering’, as Ahmed suggests, wherein the unsupported body becomes ‘lost, undone, thrown’.^33^ By engaging with such constitutive ruptures, however, *Three* brings to the fore two important polarities in queer discourse: as Heather Love describes, both the life-affirming, ‘utopian desires’ of the queer position, and the ‘traumatic dimensions’ located within queer history and queer texts, namely darkness, ambivalence, pain and loss.^34^ Loss appears everywhere in *Three*’s form, including the repetitive gaps between line breaks and paragraphs. Loss is also inscribed in the portrayal of S’s abortion, and while *Three* is undoubtedly a ‘story of triangulation; tripartite sexual desire, jealousy and transgression’, as Jordan observes, it is the momentary ‘loss’ of Leonard from the plot that makes room for a queer tenderness to arise between Ruth and S.^35^

Firstly, S’s disappearance intensifies the gaps in her notebooks. The structural lacunae function as queer subtext, settling between what is written and guiding the reader towards what is suggested. S is caught in a web of feeling, the indeterminacy of which is portrayed in stark contrast with the explicit temporal referent:

Monday

A recognisable nausea provokes the desire to become something in their lives, anything.

Everything. But whose move is the next?^36^

In this instance, S’s feeling of being out-of-place—or rather, wanting to ‘enter’ a place where her ‘entering’ constitutes a nonnormative desire—is implied both affectively and somatically; her nausea indicates the discomfort of queer feelings, and ‘discomfort is a feeling of disorientation’.^37^ If we are to consider, as Ahmed does, heteronormative comfort as ‘an encounter between more than one body’, a seamless ‘fitting in’ of body into world, then S’s pervasive search for ‘which piece fits in precisely where’ is revealed as a fundamentally queer question, one that delineates her out-of-place-ness, her disorientated worldview.^38^ Ahmed argues that ‘to follow the rules of heterosexuality is to be at ease in a world that reflects back the couple form one inhabits as an ideal’.^39^ S’s explicit unease, then, is symptomatic of desires that transgress the ‘arrangement of couple: man/woman’ ideal.^40^ S’s inner world does not reflect the shape of the exterior world’s conventions, and this disjuncture is established in Quin’s manipulation of text and space.

The text’s gaps also represent S’s inward-facing silences, which appear comparatively queer alongside Ruth and Leonard’s incessant, unpunctuated dialogue without paragraph spacing, steeped in the quotidian. S rhetorically questions how she can ‘explain, reveal all, arrive at some kind of clarity’ and thereby ‘supplant these huge areas of unspoken thoughts’.^41^ Viewing this contrast through Ahmed’s lens, it is plausible that S’s gaps are indicative of a ‘loss’ that ‘is not empty or waiting’ but is ‘an object, thick with presence’.^42^ This technique of evoking absence-with-presence carries through to S’s recollections of Ruth, which continue over a series of line breaks. Their structure reflects the medium of a tape recording, suggesting lapses of silence, or the pause of breath:

The fifth day she stayed in bed. Curtains drawn. Radio on. Just have a bad period. She said. Oh are you going out? Do stay with me. An orange light interior of some exotic flower hovered over walls. Smell of heavy perfume. Bodies. Hers. Only the shape moulded from the sheet. Will you brush my hair? Long thick over shoulders. Stay with me a little longer it’s early yet. In that yellow enclosure. Sipping whisky and ice.^43^

Quin’s paratactic syntax, broken up by full-stops, emphasizes the intensity of their interaction, acquiring the effect of an accretion of sensorial fragments. Read in light of Leonard’s deviant orchids, the description of the room as an ‘interior of some exotic flower’ implies a homoerotic relationship between Ruth and S.^44^

The act of brushing Ruth’s hair is suggestive of this, too, alongside the close proximity to the shape of her body and the scent of her perfume, corporeal signifiers that contradict Ruth’s strained exercise of heteronormativity. Indeed, S later remarks that ‘a certain intimacy sprang up between [them], that somehow never exists when L is around’.^45^ Butler reads this ‘queer affection’ as akin to water, following the verb ‘sprang’, and notes where their sexual orientation ‘has flipped or moved off-kilter, the fluid excesses of their desire causing new ripples’.^46^ S continues:

I found myself wishing he would remain away for longer. As if R plays a role when he is with us. Except I wonder if it is not a certain role she plays with me, when we are on our own.^47^

These encounters reiterate Quin’s oscillations between pretence and sincerity, a strategy that fosters an open-ended questioning of the unresolved relationship between Ruth and S. Ruth later confesses her life is shaped by way of ‘always holding back’, because ‘things had to be relatively either black or white’, or what might be considered heterocentric.^48^ The resulting loss of connection Ruth feels is depicted as a rupture in her own sense of time; she asks of herself what ‘day, night did [she] feel this appalling separation, a certain loss of identity’.^49^ Her melancholic turn backwards is symptomatic of the oppressive nature of conformity in that she loses touch with herself. Both characters’ understated attachments and conflicted yearnings can be directly associated with the idea of disorientation as the uncertainty of direction, for, in Leonard’s absence, they remain uncertain of their own orientations towards themselves and each other.

A further example of queer, temporal rupture is the absence of children in the narrative, particularly the allusion to S’s abortion, but also the childless nature of Leonard and Ruth’s marriage. If readers are to consider, as Lee Edelman does, the child as a symbol of ‘reproductive futurism’, whereby it denotes the social order that compels its subjects to reify the fantasy of heterosexual futurity, then any subjects who actively work *against* this fantasy participate in the project of queerness.^50^ In *Three*, S’s memory of her abortion is fragmented, thus the precise time of its occurrence is unclear:

Injected made ready. In white. Like half drunk. Must not lose consciousness. Various instruments. Wad of blood retained shape of gynaecologist’s finger. Low voices in an ante-room. Cylindrical light above. Chromium. Breathe deeply. Push. Where is it – was it big enough to see? Three months. What do they do with it in a bottle throw away?^51^

Quin’s clipped syntax and spontaneous line breaks interrupt prosaic linearity, further stylistic iterations of disorientation. Although S is asked whether she would ‘like to help a childless couple’, she is firm in her choice to abort.^52^ Borrowing Edelman’s terms, S’s abortion is queer precisely because ‘queerness names the side of those *not* “fighting for the children”’.^53^ Abortion inscribes within the text a double-bind of queerness, whereby S resists both her own potential for childbearing and the opportunity for a childless couple to become a family unit. In doing so, she negates the legitimacy of familial futurity, troubling the heterocentric balance that reproductive time is so reliant upon.

## Disorientation’s Utopian Dreams

Edelman’s theory that a turn towards the future is ‘merely extending heteronormativity’s reach’ is valuable for offering a queering of abortion, but it provides little optimism for imagining a queer telos.^54^ The project of uncovering Quin’s queer temporalities, then, does not cease with Edelman’s position; Quin employs radical strategies that body forth the kind of disorientation that opens up new possibilities. Halberstam aligns queerness’ appeal with its ‘potential to open up new life narratives and alternative relations to time and space’, which reflects precisely Quin’s oeuvre.^55^ Moreover, Halberstam posits that queer subcultures generate their own temporalities, encompassing futures beyond ‘paradigmatic markers of life experience’.^56^ In *Three*, it is the reader’s responsibility to undress its inventive temporalities, the subterranean places in which queerness is located, and to orient themselves towards its uncertain terrain.

Establishing disorientation’s utopian dimension in *Three* means rooting it in figurative representations of dreams, reveries, afterthoughts, hallucinations and the subconscious, all of which are vibrant, exploratory and ‘never the same pattern no matter how many times’.^57^ In spatiotemporal terms, the locus of daydreaming is in its dislocation, and the manner in which it breaks away from linearity. Quin’s methods of montage and indeterminacy engender worlds that exist beyond the ordinary time of the narrative. In this way, Quin constitutes a new (queer) reality by means of stringing together the hypothetical with the actual. S remarks that ‘all seems now a dream, attempts at piecing together, and other dreams are only remembered’, destabilizing the indices of reality embedded within the narrative.^58^ The act of reorganization, an attempt to derive meaning from confounding feelings, is a theme that recurs:

How begin to find a shape – to begin to begin again – turning the inside out: find one memory that will lie married beside another for delight? Seems beyond attainment.^59^

Quin embarks on this attempt to turn language inside-out by way of shaping it obliquely, pressing against methodological conventions until they warp to hold her prose. By disturbing the stability of meaning, she carves out paths towards a queer future, perhaps towards one that Muñoz imagines as a ‘horizon imbued with potentiality’.^60^ S, as the most explicit marker for queerness, embodies this horizon; her existential questioning of ‘when did [she] begin to construct a moment ago or a space as between waking and dreaming’ survives even beyond her death.^61^

As a result, envisaging a queer future is also about encountering queer survival, which Benjamin Bateman argues is not simply about the extension of life, but the ‘distension of life, and feelings of aliveness’.^62^ This type of survival eschews individuality; rather ‘the self expands by losing and lives by dying’.^63^ Quin’s text operates at the strokes of fracture; her kaleidoscopic trajectory gathers momentum as characters grapple with desire and deviancy. And yet, what is left at Quin’s scenes of disorder, at the circuitous beginning and end that constitute S’s death, is the birth of a queer worldview. Quin’s determination to reposition boundaries survives, making concrete Muñoz’s demand that ‘we must dream and enact new and better pleasures, other ways of being in the world, and ultimately new worlds’.^64^ Disorientation, then, is complicit in the act of world-making; it has a profoundly utopian core.

In her notes to *Queer Phenomenology*, Ahmed writes that ‘orientation for [her] is about how the bodily, the spatial, and the social *are entangled*’.^65^ *Three* is also an entanglement of sorts, allowing objects and temporalities and affects to converge in a tapestry of interdependent relations, all participating in a process of becoming something new. Loraine Morley describes *Three* as a narrative enacting the concept of ‘becoming woman’, but it synonymously enacts a becoming queer.^66^ Quin’s polysemic strategies of subversion contribute to the disorientating dimensions of queer experience by harnessing the complexities of time and space, and their susceptibility to the cadences of feeling; with *Three*’s bemusing scaffolding, one can only attempt to gain a foothold as it swings. *Three* asks ‘which piece fits in precisely where?’ but as an opening to sustain, rather than resolve, this query.^67^ Critics who are interested in Quin remain open to ambivalence, which operates as a kind of generosity towards the ideas and directions her work beckons. Beyond Quin, this kind of critical space permits experimental writing to play and fail, mystify and elude, where disorientation offers freedom from rules while claiming a different perception of how things might be pieced together. In constructing a text against straight time and its objects, Quin’s prose glances at new forms of queer existence. Her readers concur that there is much pleasure to be found in that which veers off course.

## Footnotes

1 Quin, ‘Landscape with Three-cornered Dances’, p. 8.

2 Williams, ‘(Re)turning to Quin’, p. 10.

3 White, ‘“Turning her Over in the Flat of my Dreams”’, p. 128.

4 Guy, ‘Ann Quin on Tape’, pp. 73–92.

5 Butler, ‘“Have You Tried It with Three?”’, p. 103.

6 Smith, ‘Introduction’, p. xiii.

7 I note here that I interpret Quin’s claims in interviews as her experience of being bisexual, but this is a personal view that I hold (and is open to debate).

8 Kilian, ‘Maureen Duffy’, p. 232; Gutkin, ‘Brigid Brophy’s Aestheticism’, p. 73.

9 Quin, *Three*, p. 41.

10 Ahmed, *Queer Phenomenology*, p. 3.

11 Ahmed, *Queer Phenomenology*, p. 3.

12 Quin, *Three*, p. 3; Butler, ‘“Have You Tried it With Three?”’, p. 100.

13 Philip Stevick, ‘Voices in the Head’, p. 235.

14 Quin, *Three*, p. 54.

15 Quin, *Three*, p. 97.

16 Quin, *Three*, pp. 11–2.

17 Goody, *The Culture of Flowers*, p. 298.

18 Goody, *The Culture of Flowers*, p. 313.

19 Halberstam, ‘Theorizing Queer Temporalities’, p. 182.

20 Quin, *Three*, p. 66.

21 Quin, *Three*, p. 136.

22 Quin, *Three*, p. 9.

23 Quin, *Three*, p. 9.

24 Jordan, *Late Modernism and the Avant-Garde British Novel*, pp. 152–3.

25 Powell, ‘Ann Quin, Object Relations’.

26 Laing, *The Divided Self*, p. 43.

27 Merleau-Ponty, *The World of Perception*, p. 50.

28 I borrow the term ‘heterocentric’ from Smith’s usage in *The Queer Sixties*.

29 Ahmed, *Queer Phenomenology*, p. 158.

30 Quin, *Three*, p. 53.

31 Muñoz, *Cruising Utopia*, p. 49.

32 Muñoz, *Cruising Utopia*, p. 49.

33 Ahmed, *Queer Phenomenology*, p. 157.

34 Love, *Feeling Backward*, p. 3.

35 Julia Jordan, ‘The Quin thing’.

36 Quin, *Three*, p. 60.

37 Ahmed, *The Cultural Politics of Emotion*, p. 148.

38 Ahmed, *The Cultural Politics of Emotion*, p. 148; Quin, *Three*, p. 69.

39 Ahmed, *The Cultural Politics of Emotion*, p. 147.

40 Ahmed, *The Cultural Politics of Emotion*, p. 147.

41 Quin, *Three*, p. 61.

42 Ahmed, *Queer Phenomenology*, p. 158.

43 Quin, *Three*, p. 112.

44 Quin, *Three,* p. 112.

45 Quin, *Three*, p. 141.

46 Butler, ‘Have You Tried It With Three?’, p. 95.

47 Quin, *Three*, p. 142.

48 Quin, *Three*, p. 124.

49 Quin, *Three*, p. 124.

50 Edelman, *No Future*, p. 2.

51 Quin, *Three*, p. 38.

52 Quin, *Three*, p. 37.

53 Edelman, *No Future*, p. 3.

54 Doyle, ‘Blind Spots and Failed Performances’, p. 28.

55 Halberstam, *In a Queer Time and Place*, p. 2.

56 Halberstam, *In a Queer Time and Place*, p. 2.

57 Quin, *Three*, p. 71.

58 Quin, *Three*, p. 139.

59 Quin, *Three*, p. 56; this passage is of course reminiscent of Gertrude Stein’s essay, ‘Composition as Explanation’ (1926), in which she writes, ‘beginning again and again and again explaining composition and time is a natural thing’.

60 Muñoz, *Cruising Utopia*, p. 1.

61 Quin, *Three*, p. 53.

62 Benjamin Bateman, *The Modernist Art of Queer Survival*, p. 4.

63 Bateman, *The Modernist Art of Queer Survival*, p. 4.

64 Muñoz, *Cruising Utopia*, p. 1.

65 Ahmed, *Queer Phenomenology*, p. 181.

66 Morley, ‘The Love Affair(s) of Ann Quin’, p. 132.

67 Quin, *Three*, p. 69.
